# TNF-α, IL-1B and IL-6 affect the differentiation ability of dental pulp stem cells

**DOI:** 10.1186/s12903-023-03288-1

**Published:** 2023-08-11

**Authors:** Sema Sonmez Kaplan, Hesna Sazak Ovecoglu, Deniz Genc, Tunc Akkoc

**Affiliations:** 1 Department of Endodontics, Faculty of Dentistry, Biruni University, 10. Yıl Caddesi Protokol Yolu No: 45, 34010 Topkapı, Istanbul, Turkey; 2 Faculty of Dentistry Department of Endodontics, Marmara University, Istanbul, Turkey; 3 Department of Pediatric Health & Diseases Faculty of Health Sciences, Muğla Sıtkı Koçman University, Mugla, Turkey; 4 Research Laboratories Center, Immunology and Stem Cell Laboratory, Muğla Sıtkı Koçman University, Mugla, Turkey; 5 Immunology Department, Marmara University Medical Faculty, Istanbul, Turkey

**Keywords:** Dental pulp stem cells, Inflammatory cytokines, Osteogenic differentiation, Tumor necrosis factor-α, Interleukin-1β, Interleukin-6

## Abstract

**Background:**

This in vitro study examined the effect of the inflammatory cytokines (tumour necrosis factor-α (TNF-α), interleukin (IL)-1β, and IL-6) on osteogenic, chondrogenic, and adipogenic differentiation of dental pulp stem cells (DPSCs) which have significant relevance in future regenerative therapies.

**Methods:**

DPSCs were isolated from the impacted third molar dental pulp and determined with flow cytometry analysis. DPSCs were divided into into 5 main groups with 3 subdivisions for each group making a total of 15 groups. Experimental groups were stimulated with TNF-α, IL-1β, IL-6, and a combination of all three to undergo osteogenic, chondrogenic, and adipogenic differentiation protocols. Next, the differentiation of each group was examined with different staining procedures under a light microscope. Histological analysis of osteogenic, chondrogenic, and adipogenic differentiated pellets was assessed using a modified Bern score. Statistical significance determined using one-way analysis of variance, and correlations were assessed using Pearson’s test (two-tailed).

**Results:**

Stimulation with inflammatory cytokines significantly inhibited the osteogenic, chondrogenic and adipogenic differentiation of DPSCs in terms of matrix and cell formation resulting in weak staining than the unstimulated groups with inflammatory cytokines. On contrary, the unstimulated groups of MSCs have shown to be highly proliferative ability in terms of osteogenic, chondrogenic, and adipogenic differentiation.

**Conclusions:**

DPSCs have high osteogenic, chondrogenic, and adipogenic differentiation capabilities. Pretreatment with inflammatory cytokines decreases the differentiation ability in vitro*,* thus inhibiting tissue formation.

## Background

Stem cell-based technologies are an ideal source for regenerative medicine, immunological studies, and cell therapy because they induce tissue repair and regeneration [1, 2]. Mesenchymal stem cells (MSCs) play a key role in tissue regeneration treatment. They are rapidly adherent, clonogenic, and capable of extended proliferation in vitro [3]. In addition, they maintain stem cell properties such as self-renewal, long-term viability, and differentiation potential into mesodermal origin osteocytes, chondrocytes, and adipocytes [4, 5]. As a result of their capacity to differentiate into various cell types, MSCs play a key role in tissue and organ regeneration and have recently attracted great interest in tissue engineering [6, 7].

Even though MSCs can be isolated from many sources, such as cord blood, bone marrow, or adipose tissue [8], a very promising source is the relatively easily obtainable dental tissue. There are five types of human dental stem cells: dental pulp stem cells (DPSCs) [9], stem cells from exfoliated deciduous teeth (SHED) [10], periodontal ligament stem cells (PDLSCs) [11], dental follicle stem cells [12], and stem cells from apical papilla [13]. These MSCs express specific MSC markers, such as CD29, CD73, CD90, CD105, and CD166, and can differentiate into odontoblasts, chondrocytes, and adipocytes under appropriate circumstances [9, 10]. DPSCs can easily be isolated from the dental pulp tissue of newly extracted teeth, making the procedure relatively more straightforward and avoiding ethical dilemmas [14]. DPSCs are commonly used in the regeneration and reconstruction of dental structures in addition to bone tissue engineering after undergoing osteogenic differentiation [15, 16].

Tissue engineering techniques, including the use of MSCs, often require scaffolds and cytokines serving as inductive factors [17]. Some inflammatory cytokines alter stem cell functions as well as immune or inflammatory cells [18]. In vitro studies have revealed that cytokines can affect the differentiation process of mesenchymal progenitor cells during tissue formation. Most of these in vitro studies have used MSCs by isolating them because of their ability to adhere to plastic [19, 20].

Cytokines are commonly used superior markers of inflammation, modulating immune and inflammatory responses [21]. Tumour necrosis factor-α (TNF-α) is defined as a proinflammatory cytokine expressed in injured tissues as well as in ischaemic situations [22]. TNF-α also plays a major role in the repair process of injured tissues and promotes MSC recruitment [23–25]. Similar to TNF-α, interleukin (IL)-6 can be detected in injured tissues and stimulates osteoblast differentiation. Both TNF-α and IL-6 are released from T-cells and macrophages [26]. IL-1β plays an essential role in tissue damage and inflammation as well as cell proliferation and differentiation [27]. It also induces various metalloproteinases (MMPs), causing extracellular matrix degradation and cell migration [28, 29]. Thus, cytokines can affect MSC differentiation in addition to their role as an immune response started by injury. Kang et al. [30] and Ries et al. [31] have indicated that MSCs respond to various growth factors and cytokines. Studies have reported negative and positive effects of cytokines on the osteogenic differentiation potential of MSCs [32, 33].

Dental pulp can also express many inflammatory mediators that can combat irritants [34, 35]. Pulpal inflammation (pulpitis) increases with the progression of carious lesions [36]. Caries bacterial antigens evoke proinflammatory cytokines in various amounts [37]. Lipoteichoic acid (LTA), an amphiphilic molecule produced in large amounts by cariogenic bacteria, activates the innate immune system and induces proinflammatory cytokines such as TNF-α, IL-1, IL-8, and IL-12 [38]. IL-6 and IL-1β are also secreted when dental pulp cells are challenged with Gram-positive bacteria. In the later stages of pulpitis, IL-6 becomes a critical component due to the increase of B cells [39]. Releasing these mediators in the dental pulp triggers a series of inflammatory events, resulting in innate repair with the help of immune cells, protease inhibitors and other molecules [40]. The present study also supports that the application of DPMSCs in the inflammatory niche may transform the MSCs into a phenotype of suppression of inflammation rather than tissue regeneration.

Many in vitro studies [41–44] have evaluated the roles of inflammatory cytokines in osteogenic and chondrogenic differentiation of MSCs. However, no study has reported the role of inflammatory cytokines in the adipogenic, chondrogenic or osteogenic differentiation of DPSCs. The present study examined the effect of TNF-α, IL-1β, and IL-6 on the osteogenic, chondrogenic, and adipogenic differentiation of DPSCs in vitro. The findings can provide a better understanding of in vitro differentiation of cytokine-stimulated DPSCs and may develop the possible usage of autologous transplantation of DPSCs.

## Materials and methods

### Isolation of stem cells and DPSC culture

DPSCs were isolated from the impacted third molar teeth of three 22–30-year-old patients in the Marmara University Faculty of Dentistry Oral and Maxillofacial Surgery Department, Istanbul, Turkey. All patients provided informed consent, and the Ethics Committee of the Marmara University Clinical Researches in Istanbul, Turkey approved the study protocol (22.05.15–1). The extraction procedure was performed atraumatically and under sterile conditions. Extracted teeth were transported in Dulbecco’s phosphate-buffered saline (DPBS, Gibco, Grand Island, NY, USA) with 1% penicillin/streptomycin (Gibco, USA) within ice cubes within 4 h to the laboratory in the Department of Pediatric Allergy-Immunology, Marmara University Research Hospital, where all laboratory work was performed. The pulp was separated from the tooth by cracking the crown under sterile conditions. First, the pulp was broken down to 0.1–0.5-mm pieces mechanically with a sterile scalpel and then enzymatically treated with 2 mL of collagenase type I solution (3 mg/mL) (Gibco, USA); then, incubation for 45 min at 37 °C was carried out to digest the pulp tissue enzymatically. The enzymatic activation stopped with 2 mL of 1% penicillin/streptomycin and 10% fetal bovine serum (FBS) containing Dulbecco’s modified Eagle medium (DMEM, Gibco, USA), followed by centrifugation at 1500 rpm for 5 min. The supernatant was aspirated, and cell pellets were obtained and then suspended with 5 mL of DMEM and cultivated in T-75 flasks with a 5% CO_2_ atmosphere under 37 °C for 7 days. The culture medium was changed every 2–3 days until the cells became confluent at 80%. Thereafter, adherent cells were cultured until the third passage to characterize and analyse specific surface markers. The third passage cells are used in the culture studies. These specific cellular determinations and analyses were performed using flow cytometry.

### Flow cytometry analysis

The cells from the third passage were used to analyse cell surface antigen expressions. Approximately 1 × 10^6^ cells were counted and homogenised in PBS and incubated with antibodies at room temperature in the dark for 15 min. After incubation, 0.1% sodium azide containing PBS was added and procedure followed by centrifugation at 1200 rpm for 5 min and the cell suspension analysed with FACSCalibur Flow Cytometry device with BD Cell Quest TM software (BD Biosciences, San Jose, CA, USA). CD29, CD105, CD146, CD73, and CD90 were determined as positive antibodies, whereas CD3, CD4, CD20, CD34, CD45, and HLA-DR were determined as negative antibodies.

### Stimulation of DPSCs with inflammatory cytokines

After determining DPSCs with flow cytometry analysis for specific surface markers, DPSCs from three impacted third molar teeth were randomly divided into 5 main groups with 3 subdivisions for each group making a total of 15 groups. One group of unstimulated control and three groups treated with TNF-α (100 ng/mL) (R&D Systems, UK), IL-1β (100 ng/mL) (R&D Systems UK), or IL-6 (100 ng/mL) (R&D Systems UK) for 48 h [45].

### Differentiation of DPSCs

After culturing DPSCs with inflammatory cytokines, each group was divided into three subgroups with approximately 100,000 cells to induce osteogenic, adipogenic, and chondrogenic differentiation, leading to 12 separate differentiation groups in total. The cells were incubated in a 5% CO_2_ atmosphere under 37 °C for 7 days until they became 80%–90% confluent. For osteogenic (MesenCult, Stemcell Technologies, North America), adipogenic, and chondrogenic (Gibco, Grand Island, USA) differentiation, human MSC functional identification kits were used. For the differentiation procedure, the cells were plated in 6-well plates, and the differentiation medium was prepared following the manufacturer’s instructions. The differentiation medium changed every 3 days, and at the end of 21–28 days, the formed tissues were determined using different staining procedures.

### Staining protocols and determination of differentiation

After 28 days, osteogenic, chondrogenic, and adipogenic differentiations were determined by staining with Alizarin red, Alcian blue, and oil red, respectively. Staining solutions were prepared according to the manufacturer’s instructions, and after all groups were examined by the biological light microscope under 20 × objective. Histological analysis of osteogenic, chondrogenic, and adipogenic differentiated pellets was assessed following staining protocols using a modified version of the Bern Score proposed by Grogan et al. [46]. In brief, cell pellets were assessed using the following criteria: uniformity and intensity of staining and distance between cells/amount of matrix produced and cell morphology. Each of these three categories was scored from 0 to 3 (Table 1). The evaluation was performed by calculating the arithmetical means of the scores within three criteria, as given in Table 1.

**Table 1:** Scoring categories for osteogenic, chondrogenic and adipogenic stimulated pellets in the monolayer culture system

**Scoring categories**		**Scores**
**A. Uniformity and darkness**		
No stain		**0**
Weak staining of poorly formed matrix		**1**
Moderately even staining		**2**
Even dark stain		**3**
**B.Distance between cells/Amount of matrix accumulated**		
**Osteogenic**	No osteogenic colonies/No calcium deposit	**0**
	No osteogenic colonies/Weak staining of calcium deposits	**1**
	Osteogenic colonies/Waek staining of calcium deposits	**2**
	Osteogenic colonies/High amount of calcium deposits	**3**
**Chondrogenic**	Low cell densities with no matrix between cells	**0**
	Low cell densities with little matrix between cells	**1**
	Moderate cell density with matrix	**2**
	High cell density with moderate distance between cells	**3**
**Adipogenic**	No adipocytes/No oil droplets	**0**
	No adipocytes/Weak staining of oil droplets	**1**
	Adipocytes with weak staining of oil droplets	**2**
	Adipocytes with high amount of oil droplets	**3**
**C. Cell morphologies represented**		
**Osteogenic**	Condensed/necrotic/pycnotic bodies	**0**
	Spindle/fibrous	**1**
	Mixed spindle/fibrous with calcium deposits	**2**
	Majority calcium deposits/osteogenic	**3**
**Chondrogenic**	Condensed/necrotic/pycnotic bodies	**0**
	Spindle/fibrous	**1**
	Mixed spindle/fibrous with cartilage forming	**2**
	Majority cartilage forming/chondrogenic	**3**
**Adipogenic**	Condensed/necrotic/pycnotic bodies	**0**
	Spindle/fibrous	**1**
	Mixed spindle/fibrous with oil droplets	**2**
	Majority oil droplets/adipogenic	**3**

### Statistical analyses

Statistical analyses were performed using IBM SPSS v22 (IBM SPSS, Turkey). Statistical significance was determined using one-way analysis of variance. Correlations were assessed using Pearson’s test (two-tailed). *P* < 0.05 was set as statistically significant.

## Results

### DPSC isolation and characterisation

**Fig. 1:**
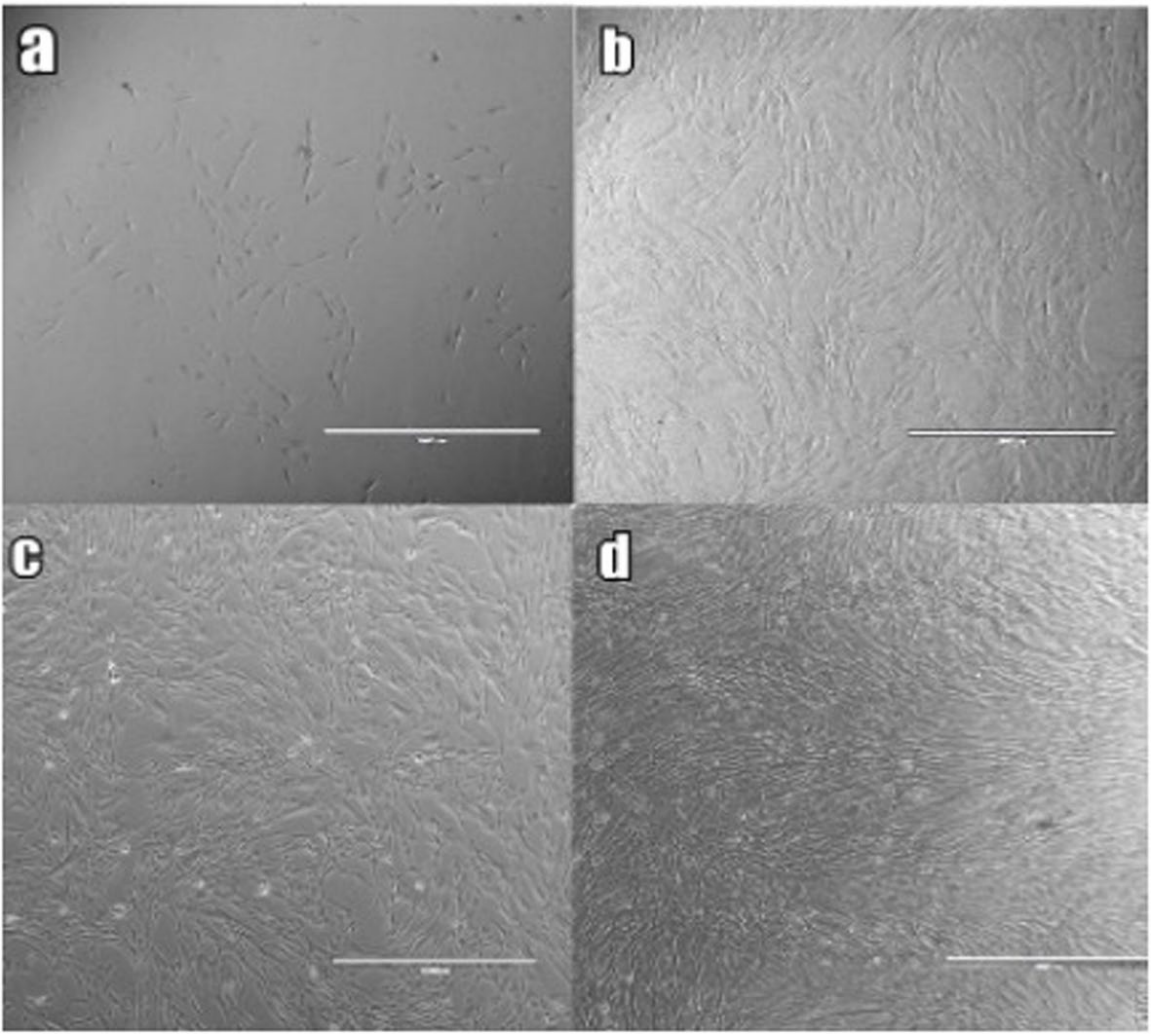
Morphological appearance of DPSCs (**a**) P0: 3^rd^ day, (**b**) P1:3^rd^ day, (**c**) P2: 3^rd^ day, (**d**): P3:3.^rd^ day. Original magnifications × 10

**Fig. 2:**
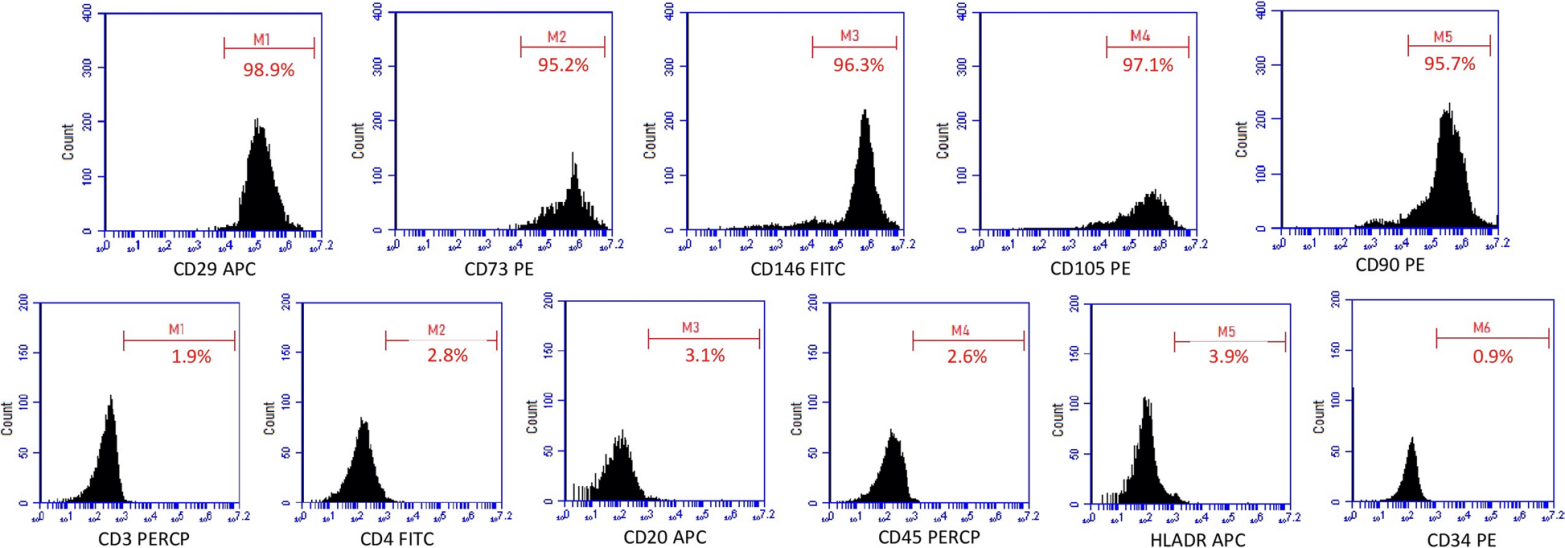
Representative flow cytometry analysis of cell surface markers on DPSCs in P3

DPSCs attached to the bottom of the culture flasks and showed a fibroblast-like morphology at the early days of incubation (Fig. 1(a)). DPSCs began to proliferate in 4–5 days and slowly formed colonies (Fig. 1(b)). Ten days after being plated in their very first cell passage, the DPSCs showed 70% confluency. Almost all the DPSCs showed a fibroblast-like spindle-shaped morphology in their later passages (Fig. 1(c), (d). Flow cytometry analysis revealed that DPSCs were positive for CD29, CD146, CD105, and CD73 and negative for CD3, CD4, and CD20 (Fig. 2).

### Osteogenic differentiation

**Fig. 3:**
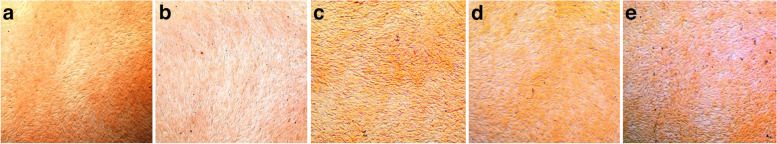
Alizarin red staining of osteogenic induced DPSCs. **a** Unstimulated. **b** Stimulated with TNF-α. **c** Stimulated with IL-1β. **D** Stimulated with IL-6. **e **Stimulated with TNF-α, IL-1β and IL-6

The unstimulated DPSC control group exhibited even, dark staining with osteogenic colonies and the highest amount of Ca^++^ deposits (Table 2, Fig. 3(a)). The TNF-α-stimulated group exhibited weak staining of the poorly formed matrix, no osteogenic colonies, and calcium deposits with spindle/fibrous cell morphology (*P* < 0.0001) (Table 2, Fig. 3(b)). The IL-1β-stimulated group exhibited moderately even staining, no osteogenic colonies with weak staining of calcium deposits, and mixed spindle/fibrous cell morphology with calcium deposits. (*P* < 0.05) (Table 2, Fig. 3(c)). The IL-6-stimulated group exhibited weak staining of the poorly formed matrix with osteogenic colonies, weak staining of calcium deposits, and spindle/fibrous cell morphology (*P* < 0.01) (Table 2, Fig. 3(d)). The DPSC group stimulated with all three cytokines showed weak staining of the poorly formed matrix with no osteogenic colonies, weak staining of calcium deposits, and spindle/fibrous cell morphology (*P* < 0.001) (Table 2, Fig. 3(e)). Stimulation with inflammatory cytokines significantly inhibited the osteogenic differentiation of DPSCs (*P* < 0.05) (Fig. 6(a)).

**Table 2:** The arithmetical means of the scores of osteogenic, chondrogenic and adipogenic stimulated pellets in the monolayer culture system according to modified version of the Bern Score

	**Osteogenic Differentiation**	**Chondrogenic Differentation**	**Adipogenic Differentiation**
**DMEM**	2,9 ± 0,2	2,9 ± 0,1	2,7 ± 0,3
**TNF-α**	0,6 ± 0,3	1,3 ± 0,3	1,2 ± 0,2
**IL-1β**	1,9 ± 0,3	0,3 ± 0,2	1,6 ± 0,1
**IL-6**	1,6 ± 0,2	0,6 ± 0,3	1,7 ± 0,3
**Mix**	0,7 ± 0,4	0,4 ± 0,2	0,8 ± 0,2

### Chondrogenic differentiation

**Fig. 4:**
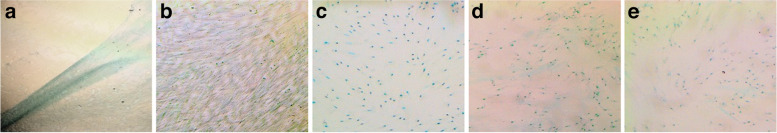
Alcain blue staining of chondrogenic induced DPSCs. **a** Unstimulated. **b** Stimulated with TNF-α. **c** Stimulated with IL-1β. **d** Stimulated with IL-6. **e** Stimulated with TNF-α, IL-1β and IL-6

The unstimulated DPSC control group exhibited even, dark staining with high cell density, moderate distance between cells, and majority cartilage forming/chondrogenic cell morphology (Table 2, Fig. 4(a)). The TNF-α-stimulated group exhibited moderately even staining, low cell density with little intercellular matrix, and spindle/fibrous cell morphology (*P* < 0.001) (Table 2 Fig. 4(b)). The IL-1β-stimulated groups showed weak staining of the poorly formed matrix, low cell density with no intercellular matrix, and condensed/necrotic bodies (*P* < 0.0001) (Table 2, Fig. 4(c)). The IL-6-stimulated group exhibited weak staining of the poorly formed matrix, low cell density with little intercellular matrix, and condensed/necrotic cell bodies (*P* < 0.0001) (Table 2 Fig. 4(d)). The DPSC group stimulated with all three cytokines showed weak staining of the poorly formed matrix, low cell density with no intercellular matrix, and condensed/necrotic cell bodies (*P* < 0.0001) (Table 2, Fig. 4(e)). Stimulation with inflammatory cytokines significantly inhibited the chondrogenic differentiation of DPSCs (*P* < 0.05) (Fig. 6(b)).

### Adipogenic differentiation

**Fig. 5:**
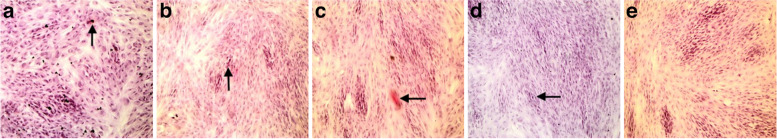
Oil Red staining of adipogenic induced DPSCs. **a** Unstimulated. **b** Stimulated with TNF-α. **c** Stimulated with IL-1β. **d** Stimulated with IL-6. **e** Stimulated with TNF-α, IL-1β and IL-6. Black arrows indicate oil droplets

**Fig. 6:**
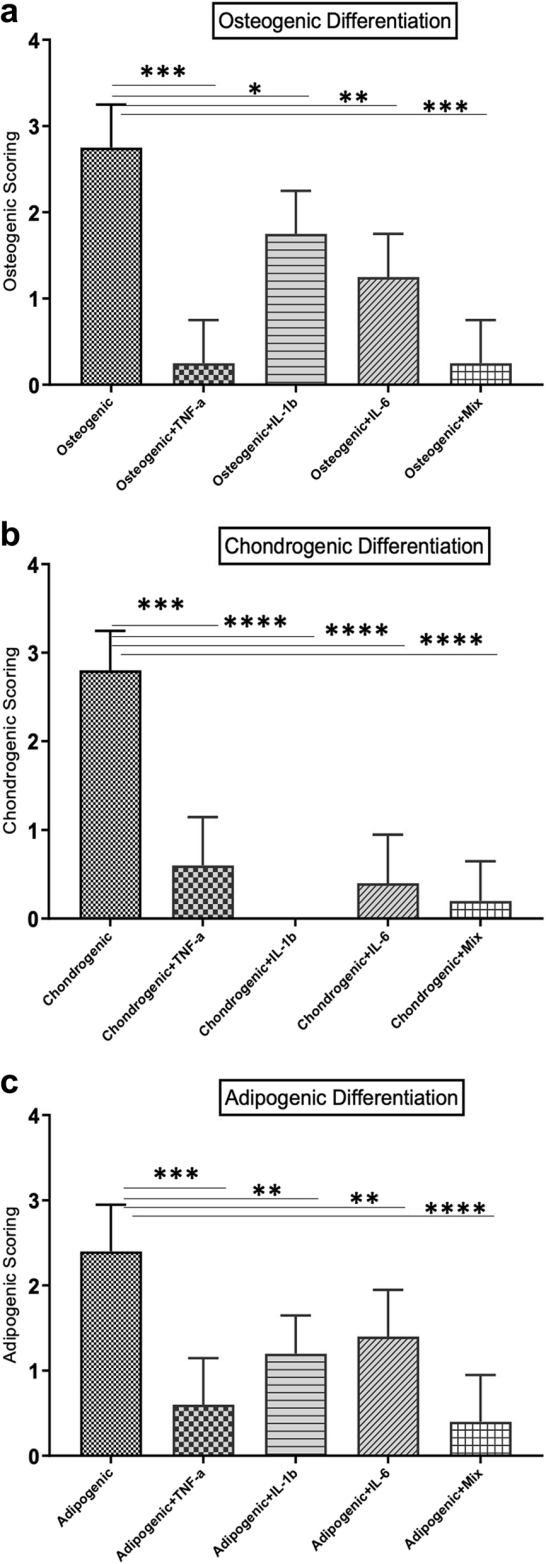
Histological scoring (Bern Score). Results are presented as mean ± SD. **a** Osteogenic differentiation. **b** Chondrogenic differentiation. **c** Adipogenic differentiation. (*: *P* < .05, **: *P* < .001, ***: *P* < .001, ****: *P* < .0001)

The unstimulated DPSC control group exhibited moderately even staining, adipocytes with a high amount of oil droplets, and adipogenic cell morphology as most oil droplets (Table 2, Fig. 5(a)). The TNF-α-stimulated group exhibited weak staining of the poorly formed matrix, adipocytes with weak staining of oil droplets, and spindle/fibrous cell morphology (*P* < 0.001) (Table 2, Fig. 5(b)). The IL-1β-stimulated group exhibited moderately even staining, adipocytes with weak staining of oil droplets, and spindle/fibrous cell morphology (*P* < 0.01) (Table 2, Fig. 5(c)). The IL-6-stimulated group exhibited moderately even staining, adipocytes with weak staining of oil droplets, and mixed spindle/fibrous cell morphology with oil droplets (*P* < 0.01) (Table 2, Fig. 5(d)). The DPSC group stimulated with all three cytokines showed weak staining of the poorly formed matrix, no adipocytes, no oil droplets, and spindle/fibrous cell morphology (*P* < 0.0001) (Table 2, Fig. 5(e)). Stimulation with inflammatory cytokines significantly inhibited the adipogenic differentiation of DPSCs (*P* < 0.05) (Fig. 6(c)).

## Discussion

### Characterisation of DPSC via morphology and stemness marker

MSCs are a promising source of stem cells and have been isolated from various tissues with different techniques [2, 32, 47]. After Friedenstein et al. [48] described MSCs in 1968, MSCs were defined in further studies as expressing CD73, CD90, and CD105 and not expressing CD11b, CD14, CD34, and CD45. Additionally, MSCs must be adherent to plastic surfaces and able to differentiate into certain cells in vitro as osteoblasts, chondroblasts, and adipocytes [49]. In our study, we used DPSCs, which we thought had the advantage of relatively easy access. The stem cell sources were the impacted third molar teeth, which were extracted during dental health care procedures, resulting in no ethical debate. Furthermore, a large proliferation capacity, keeping up their cellular phenotype for an extended period, and promising cell lines for regenerative applications were also considered among the great advantages of DPSCs [50, 51]. The two most commonly used techniques for isolating DPSCs from dental pulp tissue are the explant method and enzymatic digestion. Hilkens et al. [52] reported no difference in the tissue differentiation potential of DPSCs regarding the isolation method. In our study, the enzymatic digestion method used to isolate DPSCs was based on previous studies [9, 10]. After isolation, the cells displayed fibroblast-shaped morphology and were adherent to plastic Petri plates. Flow cytometry analysis declared that the cells showed positive expression for CD29, CD105, CD146, and CD73 markers and negative for CD3, CD4, and CD20 markers, which agree with the criteria of the International Society of Cellular Therapy [48]. Osteogenic, chondrogenic, and adipogenic differentiation procedures were performed on characterised DPSCs, and Alizarin red, Alcian blue, and oil red stains were used, respectively, to determine their differentiation into cell lines in accordance with previous studies [2, 53, 54]. Tarte et al. [55] also used staining procedures to compare the proliferation and differentiation of SHEDs and PDLSCs, but used von Kossa staining instead of Alcian blue to determine chondrogenic differentiation. MSCs can differentiate in vitro spontaneously or by the induction of biologically active molecules [56]. DPSCs can also proliferate and differentiate, as can other stem cells of dental origin [2, 9, 10, 51].

### Effect of cytokine on differentiation

Cytokines modulate immune and inflammatory responses and are markers of inflammation [21, 57]. In many situations, certain tissues need to be regenerated due to injury. Whether the tissue injury is caused by microorganisms (e.g. pulpitis) or trauma (e.g. bone fractures), proinflammatory cytokines are the superior markers of inflammatory responses [32, 58]. Both positive and negative impacts of cytokines on MSC differentiation and tissue healing have been reported [32, 47]. The present study determined how DPSCs might behave in an inflammatory environment set up with some key proinflammatory cytokines TNF-α, IL-1β, and IL-6. Many previous in vitro and in vivo studies have evaluated their roles in osteogenic and chondrogenic differentiation of MSCs. Kondo et al. [59]. indicated that in the early stages, TNF-α, IL-1β, and IL-6 contribute to fracture healing and bone remodelling. Xie et al. also [54] demonstrated that IL-6 promotes osteogenic differentiation in BM-MSCs in vitro. In vitro studies demonstrating the effects of TNF-α, IL-1β and IL-6 have mostly involved osteogenesis with MSCs other than dental origin and have not directly compared their effects on differentiation [32, 47]. Contrary to our results, Liu et al. [51] demonstrated that TNF-α promoted the osteogenic differentiation of DPSCs in vitro. Similarly, Feng et al. [25] demonstrated that TNF-α activates the NF-κB pathway and promotes osteogenic differentiation of DPSCs in vitro. Another in vitro study showed increased calcium deposits following IL-1β pretreatment when culturing BM-MSCs in osteogenic medium.

On contrary, Kondo et al. [59] also reported bone resorption can be induced under IL-6 stimulation. Lacey et al. [32] compared the effects of TNF-α and IL-1β on the osteogenic capacity of murine MSCs and found that these cytokines inhibited MSC differentiation to osteoblasts, which agrees with our findings. Liu et al. [51] investigated osteogenic differentiation of DPSCs promoted by TNF-α; this was similar to our study with the difference of evaluating transcriptome changes. Additionally, relatively long-term exposure to inflammatory mediators were reported to suppresses DPSC differentiation ability [60].

Considering the chondrogenic differentiation of BM-MSCs, Mumme et al. demonstrated the most intense staining for cartilage with low-dose IL-1β (10 and 50 pg/mL) [41]. The discrepancies in the outcomes among these studies and our study can be explained by the differences in the concentrations of proinflammatory cytokines and in the origin of the stem cells.

A limitation of the present study was that it was an in vitro analysis and not in vivo. The cytokine concentrations used in the study were used as the highest possible concentrations to assess the differentiation potential of DPSCs into the inflammatory niche. These concentrations may not be similar in vivo. In addition, the differentiation potential of DPSCs may vary in the presence of anti-inflammatory drugs such as anti-TNF-α, anti-IL6, which are used in some autoimmune or inflammatory diseases. Nonetheless, our findings might be useful for further studies for understanding the mechanisms and outcomes of DPSC differentiation with specific cytokine modulation both in vitro and in vivo because the functions and expressions of proinflammatory cytokines during certain tissue differentiations remain unclear in vivo [54]. In addition, stem cells of dental origin are expected to be preferred more frequently in future research because they are easy to obtain. Future studies should be designed to include different concentrations of inflammatory cytokines, evaluation of gene expression, and use of dental stem cells with different origins.

## Conclusion

Our results indicated that DPSCs are highly proliferative MSCs in terms of osteogenic, chondrogenic, and adipogenic differentiation. In the present in vitro study, TNF-α, IL-1β, and IL-6 were demonstrated to inhibit DPSC differentiation and tissue formation. Further studies, including in vivo applications with different dental MSCs origins and diverse amount, type and appliance durations are required to more comprehensively understand the underlying molecular mechanisms for application in stem cell therapies.

## Data Availability

The datasets generated during and analyzed during the current study are not publicly available due to the protocol submitted to the Ethics Committee Of X University but are available from the corresponding author on reasonable request.
